# Dynamic nuclear polarization-magnetic resonance imaging at low ESR irradiation frequency for ascorbyl free radicals

**DOI:** 10.1038/srep21407

**Published:** 2016-02-19

**Authors:** Shinji Ito, Fuminori Hyodo

**Affiliations:** 1 Innovation Center for Medical Redox Navigation, Kyushu University, Maidashi, Higashi-ku, Fukuoka 812-8582, Japan

## Abstract

Highly water-soluble ubiquinone-0 (CoQ_0_) reacts with ascorbate monoanion (Asc) to mediate the production of ascorbyl free radicals (AFR). Using aqueous reaction mixture of CoQ_0_ and Asc, we obtained positively enhanced dynamic nuclear polarization (DNP)-magnetic resonance (MR) images of the AFR at low frequency (ranging from 515 to 530 MHz) of electron spin resonance (ESR) irradiation. The shape of the determined DNP spectrum was similar to ESR absorption spectra with doublet spectral peaks. The relative locational relationship of spectral peaks in the DNP spectra between the AFR (520 and 525 MHz), ^14^N-labeled carbamoyl-PROXYL (^14^N-CmP) (526.5 MHz), and Oxo63 (522 MHz) was different from that in the X-band ESR spectra, but were similar to that in the 300-MHz ESR spectra. The ratio of DNP enhancement to radical concentration for the AFR was higher than those for ^14^N-CmP, Oxo63, and flavin semiquinone radicals. The spectroscopic DNP properties observed for the AFR were essentially the same as those for AFR mediated by pyrroloquinoline quinone. Moreover, we made a success of *in vivo* DNP-MR imaging of the CoQ_0_-mediated AFR which was administered by the subcutaneous and oral injections as an imaging probe.

Ascorbate is widely used, not only as a nutrient (vitamin C), but also as an antioxidant as it is a strong reducing agent. Metabolic reaction between ascorbate and dehydroascorbate is influenced by *in vivo* redox status. Recently, it is proposed that hyperpolarization technique of their isotope (1-^13^C) molecules with nuclear magnetic resonance (NMR) spectroscopy is used to monitor the *in vivo* redox status for clinical applications^1^,^2^. On the other hand, ascorbyl free radicals (AFR), which are monoanionic in form with an unpaired electron non-localized on the structural part with carbonyl groups, are generated by one electron oxidation of ascorbate monoanion (Asc)^3^. Asc reacts with superoxide anion radicals^4^, nitric oxide^5^, and vitamin E radicals^6^,^7^, which are involved in oxidative stress, to produce AFR. Therefore, AFR also has a potential to monitor oxidative stress as an *in vivo* redox marker, which has a merit of using normal vitamin C molecules (with no isotope labeling). For the detection of AFR, electron spin resonance (ESR) spectroscopy at high ESR frequency, such as X-band ESR spectroscopy, has been frequently used^8^ and the spectrophotometric measurement of UV absorption has also been reported^9^. *In vitro* investigations of endogenous AFR have been carried out with the X-band ESR spectroscopic detection^10^,^11^. The ESR spectroscopy with the high frequency has the high sensitivity of small amount of free radicals, but gives a disadvantage to penetration of microwave into the body for *in vivo* detection^12^,^13^.

Dynamic nuclear polarization (DNP)-magnetic resonance (MR) imaging at low ESR irradiation frequency has been developed as a new *in vivo* imaging method for free radicals^14^. This imaging method can provide accurate information pertaining to the precise anatomical location of free radicals in the body, since the images of free radicals have high intensity, high resolution, and clear edge^15^. Recently, using the chemically-synthesized free radicals, including pH- or redox-sensitive nitroxides^16^,^17^,^18^,^19^ and oxygen- or superoxide sensitive Oxo63^20^,^21^,^22^ (a triarylmethyl radical), as imaging probes, *in vivo* DNP-MR imaging has been used to investigate redox status^23^,^24^, oxygen concentrations in tumors^25^,^26^, pH levels in the stomach^27^, and mitochondrial function^28^. On the other hand, Hyodo *et al.* have previously reported *in vitro* spectroscopic DNP-MR imaging of free radical intermediates of biologically indispensable molecules, including flavin mononucleotide (FMN), flavin adenine dinucleotide, α-tocopherol, vitamin K_1_, and ubiquinone-10 [oxidized coenzyme Q_10_ (CoQ_10_)]^29^. The reported aqueous samples of the flavin semiquinone radicals seem the most likely candidates for *in vivo* imaging probes.

DNP-MR imaging at the low ESR irradiation frequency has the potential for noninvasive and real-time *in vivo* imaging of AFR for preclinical and clinical applications, such as the evaluations of oxidative stress-induced diseases and injuries^30^. It is necessary to understand the DNP properties of AFR for *in vivo* DNP-MR imaging of AFR. However, there have been no reports to date pertaining to the DNP properties of AFR. In addition, the combined DNP-MR imaging of AFR with the nitroxides and Oxo63 (likely, also the flavin semiquinone radicals) could potentially provide a multi-faceted approach for the evaluations of the diseases. Even taking into account this usage, it is important to make clear similarities and differences of the DNP properties between AFR and the well-known free radicals.

To approach the DNP-MR imaging of AFR, we focused on AFR produced in the redox cycling reaction between Asc and quinone molecules^31^. It has been reported that extremely water-soluble ubiquinone-0 [the oxidized form of coenzyme Q_0_ (CoQ_0_)] can highly mediate the production of AFR in an aqueous solution^32^,^33^. We initially selected the CoQ_0_-mediated AFR as a model of aqueous AFR sample to examine the spectroscopic DNP properties of AFR, and optimized aqueous mixtures of Asc and CoQ_0_ by changing their concentrations to produce AFR with a large amount (Figure S-1). We found that an aqueous mixture of 1250 mM Asc and 12.5 mM CoQ_0_ gave the AFR with enough amount and chemical stability (Figure S-2) to allow for the successful *in vitro* DNP-MR imaging using our system at low ESR irradiation frequency (515–530 MHz).

Using this aqueous mixture of Asc and CoQ_0_, we performed DNP-MR imaging of the CoQ_0_-mediated AFR at various ESR irradiation frequencies to determine the DNP spectrum of AFR, and compared this DNP-spectrum with each of the X-band, L-band, and 300-MHz ESR spectra of the AFR. Additionally, we examined the effects of the power and duration of the ESR irradiation on the DNP-MR imaging of the CoQ_0_-mediated AFR. Herein, we report the basic spectroscopy on DNP properties of the CoQ_0_-mediated AFR, and discuss it comparing with those of AFR produced in the similar typed reaction of Asc and pyrroloquinoline quinone (PQQ) and in the different typed reaction of Asc and Trolox radicals. Moreover, we demonstrate *in vivo* DNP-MR imaging of the CoQ_0_-mediated AFR administered subcutaneously or orally into mice.

## Results

### Determination of DNP spectrum for CoQ_0_-mediated AFR

**Figure 1:**
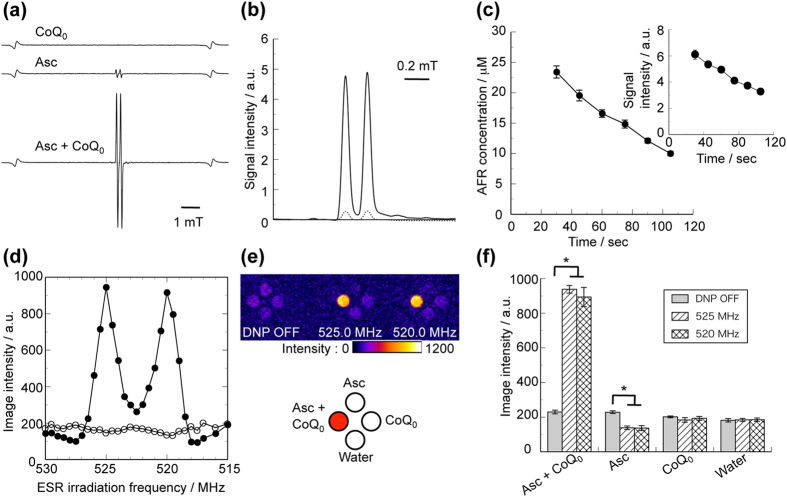
DNP spectrum of CoQ_0_-mediated AFR. (**a**) Typical X-band ESR spectra for 12.5 mM CoQ_0_ solution, 1250 mM Asc solution, and the mixture of 1250 mM Asc and 12.5 mM CoQ_0_. (**b**) X-band ESR absorption spectra for the Asc solution (dotted line) and the mixture of Asc and CoQ_0_ (solid line). (**c**) Time variations in concentration of the AFR and the signal intensities at spectral peak (inset) for the mixure of Asc and CoQ_0_. (**d**) DNP spectra for the Asc solution (open circle) and the mixture of Asc and CoQ_0_ (closed circle). DNP-MR images (**e**) and graphs of the image intensities (**f**) for the mixture of Asc and CoQ_0_, the Asc and the CoQ_0_, and ultrapure water. In (**a**), the doublet spectrum was recorded at 60 s after the mixing. In (**b**), the absorption spectra are obtained by integration of the spectra shown in (**a**). In (**c**), a horizontal axis indicates the times at which the doublet spectrum was recorded. Data are the mean ± SD (n = 6). In (**f**), data are mean ± SD (n = 6, *P < 0.001 as determined by Student’s *t* test).

We mixed aqueous solutions of 2500 mM sodium ascorbate (pH 7) and 25 mM CoQ_0_ (pH 3.5) at equal volumes to prepare the mixture of 1250 mM Asc and 12.5 mM CoQ_0_. As shown in Fig. 1a, the X-band ESR spectrum of this mixture indicated the remarkable increase of a specific doublet spectrum to the AFR with no significant production of semiquinone radicals of CoQ_0_. In the measurement of this doublet AFR spectrum recorded at 60 s after the mixing, its signal intensity was 17-fold greater than that for the original AFR in the 1250 mM sodium ascorbate aqueous solution (Fig. 1b). The AFR concentration and the pH values for the mixture of Asc and CoQ_0_ decreased from 25 to 10 μM (Fig. 1c), and from 8.4 to 8.0 (Figure S-2d), respectively, between 30 and 105 s after the mixing, which corresponds to the scan time of our DNP-MR imagings. We performed the DNP-MR imagings for the mixture of Asc and CoQ_0_, and the 1250 mM Asc solution at various ESR irradiation frequencies ranging from 515–530 MHz (Figure S-5). The intensities of the DNP-MR images for these samples are plotted with respect to ESR irradiation frequencies to obtain the DNP spectra as shown in Fig. 1d. The DNP spectrum for the CoQ_0_-mediated AFR has a clear doublet shape which is similar to the AFR spectrum in the X-band ESR absorption spectrum, in which the images are positively enhanced in the range of 518.5–526.5 MHz, indicating two peaks at 520 and 525 MHz, and are negatively enhanced at 517.5 and 527.5 MHz, and in these vicinities. The intensities of the spectral peaks at 520 and 525 MHz for the DNP-MR images of the CoQ_0_-mediated AFR were 4-fold greater those of the images obtained without ESR irradiation (DNP OFF) (Fig. 1e,f). The DNP spectrum of the 1250 mM Asc solution indicates slightly negative enhancement at 520 and 525 MHz, and in these vicinities.

### Comparison of DNP spectra for CoQ_0_-mediated AFR, ^14^N-CmP, and Oxo63

**Figure 2:**
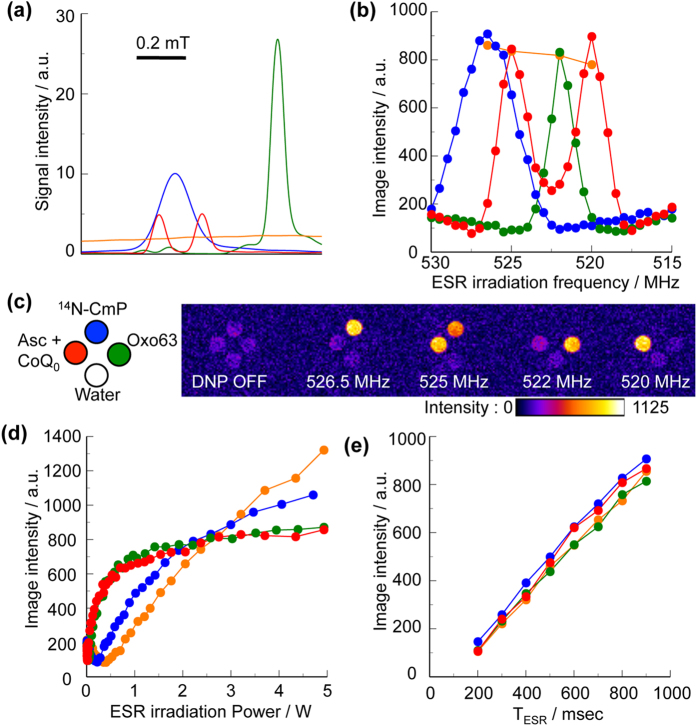
DNP spectra of CoQ_0_-mediated AFR, ^14^N-CmP, and Oxo63. X-band ESR absorption spectra (**a**), DNP spectra (**b**), representative DNP-MR images (**c**), and changes in the intensities of the DNP-MR images obtained for various ESR irradiation powers (**c**) and T_ESR_ (**d**), for the mixture of 1250 mM Asc and 12.5 mM CoQ_0_ (red), 150 μM ^14^N-CmP (blue) and 60 μM Oxo63 (green) solutions, and the mixture of 9 mM FMN and 9 mM NADH (orange). In (**a**), the doublet spectrum was recoded at 60 s after the mixing. For the mixture of FMN and NADH, in (**a**) a portion of the ESR absorption spectrum (Figure S-7b) is shown, and in (**b**) plots of the image intensities (Figure S-7d) are superimposed on Figure S-6b. The images in (**c**) are from Figure S-6a. Changes in the intensities of the DNP-MR images obtained for various ESR irradiation powers (**d**) and T_ESR_ (**e**), for the mixture of 1250 mM Asc and 12.5 mM CoQ_0_ (red), 150 μM ^14^N-CmP (blue) and 60 μM Oxo63 (green) solutions, and the mixture of 9 mM FMN and 9 mM NADH (orange).

To compare the DNP spectrum of the CoQ_0_-mediated AFR with those of ^14^N-CmP and Oxo63, the DNP-MR images for CoQ_0_-mediated AFR, 150 μM ^14^N-CmP, and 60 μM Oxo63, were simultaneously obtained at the ESR irradiation frequency in the range of 515–530 MHz (Figure S-6). The X-band ESR and DNP spectra for these samples are shown in Fig. 2a,b, respectively. A portion of the ESR spectrum and four plots showing the image intensities at the different peak positions for FMN semiquinone radicals (FMNH) sample (an aqueous mixture of 9 mM FMN and 9 mM NADH) (Figure S-7) are also indicated. The relative locational relationship between the doublet spectral peaks (520 and 525 MHz) of the CoQ_0_-mediated AFR, and the center spectral peaks of ^14^N-CmP (526.5 MHz) and Oxo63 (522 MHz), indicated the difference from that in the X-band ESR absorption spectra.

### Effect of the power and duration (T_ESR_) of the ESR irradiation

To compare the effect of the power of the ESR irradiation for CoQ_0_-mediated AFR, ^14^N-CmP, Oxo63, and FMNH, we obtained the DNP-MR images with various ESR irradiation powers until 5 W at each ESR frequency of the spectral peaks for the samples used in Fig. 2b, and plotted these image intensities with respect to the ESR irradiation power (Fig. 2d). The image intensities for the CoQ_0_-mediated AFR increased describing a curve as the power rises, and were saturated at the vicinity of 3 W. Such power-dependent saturation of AFR was also observed in the ESR spectroscopy^34^. The power-dependent curve of the DNP-MR imaging for the CoQ_0_-mediated AFR is similar to that for Oxo63. The image intensity for the AFR was saturated at the lower power of the ESR irradiation compared with ^14^N-CmP and FMNH. The difference in the power dependencies for these samples may be caused by differences in the spectral widths and the radical concentrations (Table 1). The half widths of each the spectrum for the doublet AFR spectrum are almost equal to that for 60 μM Oxo63, and are half that for 150 μM ^14^N-CmP. Also, we obtained the DNP-MR images at various T_ESR_ ranging from 0.2–0.9 s for these samples (Fig. 2e). The image intensities for all the samples increased linearly in a similar manner as the T_ESR_ rises.

### L-band and 300-MHz ESR spectra for CoQ_0_-mediated AFR

**Figure 3:**
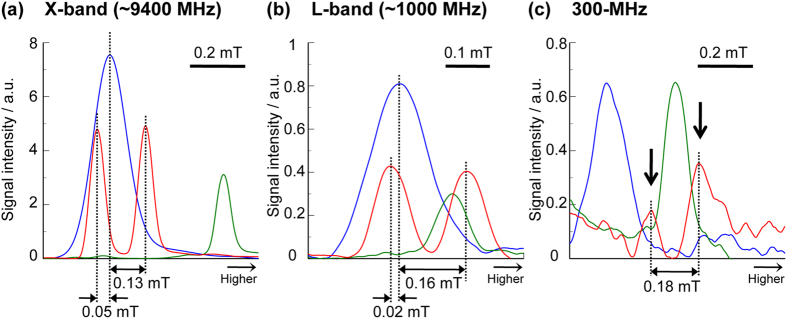
X-band, L-band, and 300-MHz ESR spectra of CoQ_0_-mediated AFR, ^14^N-CmP and Oxo63. X-band (**a**), L-band (**a**), and 300-MHz (**b**) ESR absorption spectra for the mixture of 1250 mM Asc and 12.5 mM CoQ_0_, ^14^N-CmP and Oxo63 solutions. The concentrations of ^14^N-CmP and Oxo63 solutions were 100 and 10 μM in (**a**) and (**b**), and 200 and 50 μM in (**c**), respectively. These absorption spectra are obtained by integration of the spectra shown in Figure S-3. In (**c**), spectral peaks of the AFR doublet spectrum are indicated by the arrows.

To examine the difference of the relative locational relationship of the spectral peaks in the X-band and DNP spectra for CoQ_0_-mediated AFR, ^14^N-CmP, and Oxo63, we measured the L-band (~1000 MHz) and 300-MHz ESR spectra, which are obtained with the ESR frequencies near the ESR irradiation (515–530 MHz), for these aqueous samples, and compared these spectra with the X-band ESR spectra (Fig. 3). All the spectra of the CoQ_0_-mediated AFR for these ESR spectroscopies showed the same distances of ca. 0.18 mT between the two peaks of the doublet spectrum. The distances between this two peaks and the center spectral peak of the ^14^N-CmP solution, were 0.05 and 0.13 mT in the X-band ESR spectra, and were 0.02 and 0.16 mT in the L-band ESR spectra, respectively. The L-band ESR spectra showed that the peak location of the doublet AFR spectrum relative to the ^14^N-CmP and Oxo63 solutions, shifted toward the side of the higher magnetic field, compared with that in the X-band ESR spectra. The 300-MHz ESR spectra showed the shift more remarkably.

For these ESR spectra measurements, as the range of the ESR frequency is lower, the peak locations of the doublet AFR spectrum relative to those of ^14^N-CmP and Oxo63 shifted toward the side of higher magnetic field. Little is known about the cause of this relative spectral shift, but it might be possibly related to differences in hyperfine interactions which induce the Knight shift^35^. These observations suggest that the peak location of the doublet AFR spectrum relative to ^14^N-CmP and Oxo63 depends on the ESR frequency. In agreement with this suggestion, the relative locational relationship of the spectral peaks of the DNP spectra in the range of 515–530 MHz between the CoQ_0_-mediated AFR, ^14^N-CmP, and Oxo63, was similar to those in the 300-MHz ESR spectra rather than the L-band ESR spectra.

### Determination of the DNP enhancement factor

The image intensities of the doublet spectral peaks for the CoQ_0_-mediated AFR are almost equivalent to those of the central spectral peaks for 150 μM ^14^N-CmP and 60 μM Oxo63, and to that for the FMNH sample at 525 MHz (Fig. 2b). The DNP enhancement factors for these samples were determined (Table 1). The DNP enhancement factor, ε, is defined as ε = I_z_/I_0_, where I_z_ and I_0_ are the image intensities with, and without, ESR irradiation, respectively, as described in the previous report by Hyodo *et al.*^29^. Among these samples, the CoQ_0_-mediated AFR had the highest value for the ratio of enhancement factor to radical concentration.

### Comparison of DNP spectra for PQQ-mediated AFR, ^14^N-CmP, and Oxo63

**Figure 4:**
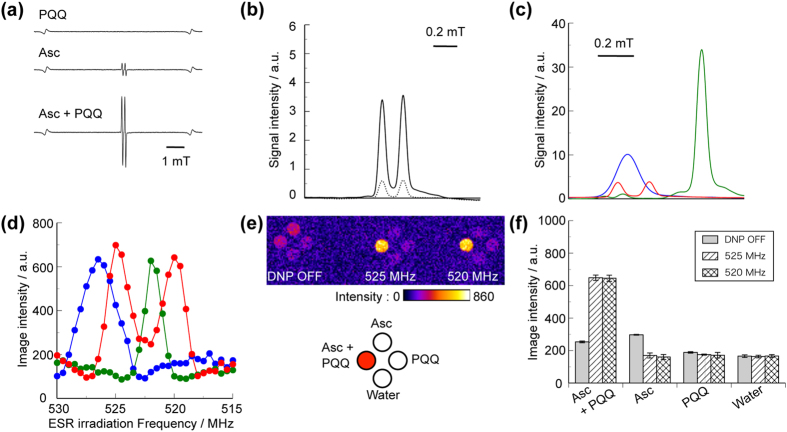
DNP spectra of PQQ-mediated AFR, 14N-CmP, and Oxo63. (**a**) Typical X-band ESR spectra for the mixture of 2125 mM Asc and 4.5 mM PQQ, 2125 mM Asc solution, and 4.5 mM PQQ solution. (**b**) X-band ESR absorption spectra for the Asc solution (dotted line) and the mixture of Asc and PQQ (solid line). X-band ESR absorption (**c**) and DNP spectra (**d**) for the mixture of Asc and PQQ (red), and 90 μM ^14^N-CmP (blue) and 50 μM Oxo63 (green) solutions. (**e**) Representative DNP-MR images without ESR irradiation (DNP OFF) or with the ESR irradiation at frequencies of 525 and 520 MHz for the mixture of 2125 mM Asc and 4.5 mM PQQ, 2125 mM Asc and 4.5 mM PQQ solutions, and ultrapure water. (**f**) Graphs showing the intensities of the DNP-MR images in (**e**). In (**a**) and (**c**), the doublet spectrum was recorded at 60 s after the mixing. In (**b**), the absorption spectra are obtained by integration of the spectra shown in (**a**). The data in (**f**) represent the mean values ± SD (n = 3).

PQQ^36^,^37^, another highly water-soluble quinone molecule^38^, is a novel vitamin-like biofactor which functions as an antioxidant^39^ and scavenger of reactive oxygen species^40^. We mixed aqueous solutions of 2500 mM sodium ascorbate and 30 mM pyrroloquinoline quinone disodium (adjusted to pH 5 with NaOH and HCl solutions) at a volume ratio of 17:3 to prepare a mixture of 2125 mM Asc and 4.5 mM PQQ. The X-band spectrum of this mixture indicated the markedly increased doublet spectrum showing AFR (Fig. 4a). In the measurement of this doublet AFR spectrum recorded at 60 s after the mixing, its signal intensity was 5.7-fold greater than that for the original AFR in 2125 mM sodium ascorbate aqueous solution (Fig. 4b). This mixture of Asc and PQQ also gave the AFR with enough amount and chemical stability to allow for the *in vitro* DNP-MR imaging with our system (Figure S-8).

Using this mixture, we obtained positively enhanced DNP-MR images of the PQQ-mediated AFR (Figure S-9). The locations of the spectral peaks in the X-band ESR and the DNP spectra for the PQQ-mediated AFR relative to those of ^14^N-CmP and Oxo63, were the same as those of the CoQ_0_-mediated AFR (Fig. 4c,d). The DNP-MR images of the PQQ-mediated AFR were obtained simultaneously with 2125 mM Asc and 4.5 mM PQQ aqueous solutions at the ESR irradiation frequencies at 520 and 525 MHz, or without ESR irradiation (Fig. 4e). The intensities of these DNP-MR images were 2.5-fold greater than those of the images obtained without ESR irradiation (Fig. 4f). The ratio of the enhancement factor to AFR concentration for the PQQ-mediated AFR was almost the same as that of the CoQ_0_-mediated AFR (Table 1).

### DNP-MR images of AFR generated by the reaction of Trolox radicals and Asc

**Figure 5:**
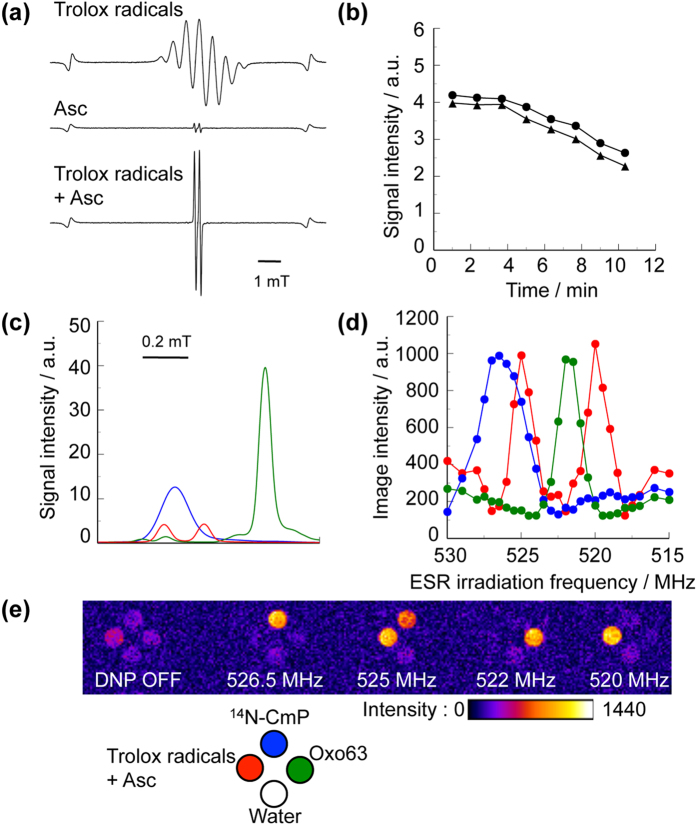
DNP-MR imaging of AFR produced by the reaction of Asc and Trolox radicals. Typical X-band ESR spectra for the Trolox radical solution, 2500 mM Asc solution, and mixture of the Trolox radical solution and 1250 mM Asc solution. X-band ESR absorption spectra (**a**), and time variations in the signal intensities of the doublet spectrum (**b**). X-band ESR absorption (**c**) DNP spectra (**d**), and representative DNP-MR images (**e**) for the mixture of Trolox radical solution and 1250 mM Asc (red), and 100 μM ^14^N-CmP (blue) and 50 μM Oxo63 (green) solutions. The images were obtained in the same conditions as those of the CoQ_0_-mediated AFR, except for, with accumulation number of 2.

We mixed the Trolox radical solution (in ethanol/18-Crown-6) with radical concentration of ca. 300 μM and 2500 mM Asc aqueous solution at equal volume to produce AFR (Fig. 5a). The AFR concentration was ca. 12 μM at 60 s after the mixing. The signal intensity of X-band ESR spectrum for this AFR indicated no significant decrease for 4 min after the mixing and started decreasing gradually after 4 min (Fig. 5b), showing relatively high chemical stability. Using this mixture, we obtained positively enhanced DNP-MR images of the AFR. The locations of the spectral peaks in the X-band ESR and the DNP spectra for the AFR were the same as those of the quinone-mediated AFR (Fig. 5c–e).

### *In vivo* DNP-MR imaging of the CoQ_0_-mediated AFR

**Figure 6:**
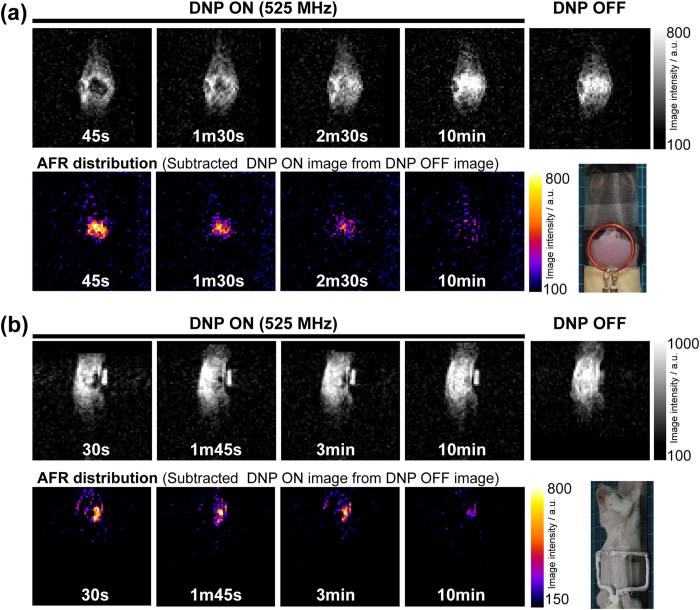
*In vivo* DNP-MR imaging of AFR. (**a**) Time variations in DNP-MR images of mouse after 500 μL of the aqueous mixture of Asc and CoQ_0_ was subcutaneously injected into the back of the mouse. The DNP-MR images were obtained by the ESR irradiation with power of 3 W (TR = 500 ms, TE = 40 ms, FA = 90 deg., T_ESR_ = 400 ms). (**b**) Time variations in DNP-MR images of mouse after 500 μL of the aqueous mixture of Asc and CoQ_0_ was orally injected into the stomach of the mouse. The DNP-MR images were obtained by the ESR irradiation with power of 8 W (TR = 1000 ms, TE = 40 ms, FA = 90 deg., T_ESR_ = 900 ms). The upper and lower images indicate original and difference DNP-MR images, respectively. The difference images are obtained by subtraction of the original images and “DNP OFF” image. Imaging times show starting times after the mixing of Asc and CoQ_0_ solutions. Injections of the mixture (for 10 sec) started in ca. 10 sec after the mixing. The start times of the scans for the imaging after the mixing are indicated.

After the mixture of Asc and CoQ_0_ was subcutaneously injected into the back of mouse or orally injected into the stomach of mouse, we performed the DNP-MR imaging of the CoQ_0_-mediated AFR with the ESR frequency of 525 MHz, and obtained enhanced DNP-MR images (upper) of the CoQ_0_-mediated AFR for both injections, as shown in Fig. 6. Subtractions of these images and the image obtained in DNP OFF give the radical images (lower). The enhanced image intensity was time-dependently decreased within 10 min.

## Discussion

We succeeded in obtaining positively enhanced DNP-MR images of AFR mediated by CoQ_0_ or PQQ at the ESR irradiation frequencies in the range of 515–530 MHz. Both the CoQ_0_- and PQQ-mediated AFR were decaying during the scan from 30–105 s, but kept enough concentration to provide positive enhancements by our imaging technique. The relative locational relationships of spectral peaks in the DNP spectra in the range of 515–530 MHz between the AFR, ^14^N-CmP, and Oxo63, were different from that in the X-band ESR spectra, but were similar to that in the 300-MHz ESR spectra. This suggests that the DNP spectroscopy of the AFR depends on the frequency of the ESR irradiation. The separation of the spectral peaks in the DNP spectra for these samples enables easy selection of the AFR spectrum in case of the presence of ^14^N-CmP and/or Oxo63.

It is noteworthy that both the ratios of enhancement factor to radical concentration for the CoQ_0_- and PQQ-mediated AFR were higher than those of the ^14^N-CmP, the Oxo63, and the FMNH samples, showing high efficiency per radical molecule for DNP of AFR. The difference of the power dependencies of the ESR irradiation between the AFR, ^14^N-CmP, and FMNH also represent an advantage for the selective DNP-MR imaging of AFR even though these DNP spectra overlap each other. For example, in Fig. 2d, the image intensities at ESR irradiation power of 0.5 and 1 W for the AFR were about twice those for 150 μM ^14^N-CmP solution and the FMNH sample, respectively. These types of characteristic information derived from the DNP spectroscopy for the AFR will give a great advantage for DNP-MR imaging of small amounts of AFR.

It is known that microwave with the frequency of 515–530 MHz can penetrate into the skin in the depth with cm level^13^. In fact, the enhanced DNP-MR images at 525 MHz ESR irradiation frequency on mice stomach and subcutaneous were obtained (Fig. 6). In our system, both processes of the ESR irradiation and the NMR signal detection are performed under the external magnetic field of 18.5 mT. Lurie *et al.* reported the field-cycle DNP-MR imaging system with rapid switching magnetic fields of 5 mT for the ESR irradiation and 450 mT for the NMR signal detection to increase the sensitivity, and obtained enhanced DNP image with clear anatomical structure^41^. Such strategy may be necessary for future clinical application, since the sensitivity of the NMR signaling increase with depending on the magnetic field.

There is a concern of generation of heat by the ESR irradiation at the higher power. For free radicals with narrow line width, such as AFR and Oxo63, it is able to obtain high enhancement with the relatively low power compared to nitroxyl radicals (Fig. 2d). In this study, there was no significant temperature increase in the phantoms and mice by the ESR irradiation with the power of 3 W (TR = 1000 ms, T_ESR_ = 900 ms) for single scan.

Utilizing our determined DNP spectrum of the AFR, we demonstrated the *in vivo* DNP-MR imaging by subcutaneous and oral injections of the CoQ_0_-mediated AFR. Although further improvement of enhancement and chemical stability of AFR may be necessary, the decay manners after exogenously administered AFR might be utilized as a maker of tissue redox status.

Our aim is to approach the *in vivo* DNP-MR imaging of not only the exogenous AFR but also endogenous AFR toward the medical application. To date, reported concentrations of the endogenous AFR are in the nanomolar range (for example, AFR levels in ischemia/reperfusion injury are about 60 nM^42^). Therefore, it is necessary to increase in the sensitivity on DNP-MRI measurement. The enhancement factor (ε) for liquid sample depends on coupling (ρ), leakage (f), and saturation (s) factors, as expressed by the formula, ε = 1 − (|γe|/γn)〉fs/n, where γe is the gyromagnetic ratio of electron spin (28.0 GHz/T), γn is the gyromagnetic ratio of nuclear spin (42.6 MHz/T for proton), and n is the number of hyperfine splittings. Enkin *et al.* mentioned that these factors are affected by the magnetic field, the ESR irradiation, and sample temperature, demonstrating that the saturation factor increased by the pulsed electron-electron double resonance^43^. Thus, improvement of the ESR irradiation and the NMR signal detection under higher magnetic field such as 1.5 or 3 T, which is widely used for clinical MR imaging systems, could increase overall sensitivity of DNP-MRI measurement.

Detailed information on the DNP properties of AFR will be necessary for such potential DNP-MR imaging of AFR. Our results are the first step toward this realization. The *in vivo* DNP-MR imaging techniques of AFR might enable the accurate identification of the specific location of oxidative stress in the body and make easy noninvasive and real-time monitoring. Consequently, it might be possible for the prevention and evaluation of oxidative stress-induced diseases and injuries at an earlier stage and with higher certainty.

In recent years, new medical applications with vitamin C have been developed, including cancer therapies based on the combined administration of vitamin C and menadione^44^ and the intravenous administration of high doses of vitamin C^45^. In these therapies, AFR are produced in reaction processes^33^,^46^,^47^, which potentially may be used to follow up distribution of the administrated medication by the *in vivo* DNP-MR imaging of the AFR. CoQ_0_ is the core structure of various biological ubiquinone molecules with isoprene units (CoQ_1_-CoQ_10_; CoQs). These ubiquinones also would react with Asc to mediate AFR. AFR mediated by the human CoQ_10_ could be possible to use for *in vivo* imaging probes. We observed that AFR production occurred in the reaction of the Asc solution and a CoQ_10_-containing liposome suspension (Figure S-10), though the AFR production was not enough for our DNP-MR imaging yet. Also, the PQQ-mediated AFR may be used for *in vivo* imaging probes with a high level of safety, considering that PQQ is water-soluble human quinone molecule.

In our experiments, we used CoQ_0_ and Trolox as water soluble molecules of CoQ_10_ and vitamin E for production of AFR by redox reaction. It is known that CoQs and vitamin E are important redox molecules and involved in mitochondrial electron transport (CoQ_10_), and in antioxidant at the biological membranes, respectively. Since the redox metabolism of AFR is affected by the localization and metabolism of these molecules, the *in vivo* DNP-MR imaging of AFR mediated by these molecules might be useful for the simultaneous monitoring of the different redox metabolism using their DNP-spectral information^23^,^29^.

The generation of more information pertaining to the DNP properties of AFR should provide further possibilities to improve the DNP-MR imaging techniques for AFR. The spectroscopic ESR investigation at low ESR frequency, such as the L-band and 300-MHz ESR, would provide the useful information to improve the DNP-MR imaging techniques as well as the *in vivo* detection by the ESR imaging for AFR. The spectroscopic properties of AFR in DNP-MR imaging can be also applied to DNP- NMR spectroscopy^48^ and the ESR spectroscopy via the DNP effect^49^. Such spectroscopic studies should contribute to new methods for the chemical analysis of AFR.

## Conclusion

We demonstrated *in vitro* DNP-MR imagings at the low ESR irradiation frequency ranging from 515–530 MHz for the AFR produced in the redox cycling reaction between Asc and water-soluble quinone molecules (CoQ_0_ and PQQ), and revealed the similarities and the differences in the spectroscopic DNP properties between the AFR and the commonly used free radicals (^14^N-CmP and Oxo63) or a candidate (FMNH) for investigations of *in vivo* DNP-MR imaging. The separation of the AFR spectrum from those of ^14^N-CmP and Oxo63 in the DNP spectra gives an advantage of selective imaging of AFR. Determination of DNP spectrum of the AFR led to the success of *in vivo* DNP-MR imaging of the CoQ_0_-mediated AFR by subcutaneous and oral injections.

## Materials and Methods

### Chemicals

Sodium L(+)-ascorbate, Ubiquinone-0 [oxidized form of coenzyme Q_0_ (CoQ_0_)] (2,3-dimethoxy-5-methyl-1,4-benzoquinone), FMN (riboflavin sodium phosphate), NADH (β-diphosphopyridine nucleotide disodium salt, reduced form), pyrroloquinoline quinone disodium salt, Trolox (6-hydroxy-2,5,7,8-tetramethylchroman-2-carboxylic acid) and 18-Crown-6 (1,4,7,10,13,16-Hexaoxacyclooctadecane) were purchased from Wako Pure Chemical Industries (Osaka, Japan). ^14^N-labeled 3-carbamoyl-2,2,5,5-tetramethyl-1-pyrrolidine-1-oxyl (carbamoyl-PROXYL) and Oxo63 (tris[8-carboxy-2,2,6,6-tetrakis(2-hydroxymethyl)benzo[1,2-*d*:4,5-*d*]bis(1,3)dithio-4-yl]methyl radical, trisodium salt) were purchased from Sigma-Aldrich Chemical Co. (Milwaukee, WI, USA) and Oxford Instruments (Tubney Woods, UK), respectively. KO_2_ was obtained from Strem Chemicals Inc. (Newburyport, MA, USA). Ultrapure (Milli Q) water was used for the preparation of aqueous solutions.

### Animals

C57/BL6 (female, 8 weeks) and ICR mice (female, 5 weeks) were purchased from Kyudo Co. (Saga, Japan). The mice were housed in a climate-controlled room with a cycle of 12 h-light and 12 h-dark cycle, and were allowed free access to water and food (MF diet, Oriental Yeast Co., Tokyo, Japan) for acclimatization to their environment for a week prior to the experiments. The cares and experimental procedures for all animals were approved by the committee on the Ethics of Animal Experiments, Kyushu University, and were conducted in accordance with the Guidelines for Animal Experiments of Kyushu University.

### X-band ESR spectroscopy

X-band ESR spectra were obtained with an X-band ESR spectrometer (model ES-FA100M, JEOL Ltd., Akishima, Japan) as described in the previous report by Hyodo *et al.*^29^. Samples (30 μL) transferred to a 100 μL-microcapillary tube (Microcaps, Drummond Scientific Company, Broomall, PA, USA) were used for measurements. The radical concentration of each radical solution was estimated from the total area [calculated as integral values, named “signal integration”, with an accessory of ESR spectrum analysis computer software (JEOL)] of the X-band ESR absorption spectra, using a standard curve indicating the relationship between radical concentrations and the total area for ^14^N-CmP. Signal intensities of the X-band ESR absorption spectra are shown with the ratio to that of a Mn^2+^ marker. The signal intensities for the doublet AFR spectrum were shown with the left side peak in the absorption spectra.

### L-band ESR spectroscopy

L-band ESR spectra were obtained with a L-band ESR spectrometer (models JES-LA2L and ES-27020, JEOL) at room temperature under the following conditions: microwave frequency of 1030 MHz; magnetic center field of 37 mT; microwave power of 10 mW; modulation width of 0.06 mT; sweep time of 20 s; sweep width of ±2.5 mT; time constant of 0.03 s. A 1-mL sample was transferred to a glass tube and used for the measurements.

### 300-MHz ESR spectroscopy

300-MHz ESR spectra were obtained with a 300-MHz ESR spectrometer (models JES-LA2L and ES-LLBA2/BU, JEOL) at room temperature under the following conditions: microwave frequency of 300 MHz; magnetic center field of 10.4 mT; microwave power of 10 mW; modulation width of 0.06 mT; sweep time of 20 s; sweep width of ±2.5 mT; time constant of 0.03 s. A 9-mL sample in a screw-capped glass bottle was used for the measurements.

### DNP-MR imaging

DNP-MR imaging was carried out by the methods described in the previous report by Hyodo *et al.* using home-made DNP-MR imaging system, in which a series of processes of the ESR irradiation and MR imaging are operated under statically fixed external magnetic fields of ~ 0.02 T. The frequency, power and duration for the ESR irradiations are changeable by modulation of a frequency synthesizer (model N9310A, Agilent Technology, Santa Clara, CA, USA) and an ESR amplifier (model N146-509AA, Thamway Co. Ltd., Fuji, Japan). We had have obtained the DNP spectra at the ESR irradiation frequency in the range of 471–537 MHz for ^14^N-CmP aqueous solution (Figure S-4).

In this study, a homemade single-turned surface coil (20-mm diameter)^15^ and a custom-made cylindrical Teflon container (18-mm o.d., 20-mm height) (Figure S-5a) were used for the ESR irradiation. A 300-μL sample of the radical solution was transferred to each of the four wells (5-mm i.d., 18-mm depth) of the Teflon container. Thus, it was possible to simultaneous obtain DNP-MR images of a maximum of four samples in a horizontal (Coronal) plane. The conditions used for a typical scan are as follows: magnetic field = 18.5 mT, MR imaging frequency = 0.793 MHz, ESR irradiation frequency = 515–530 MHz, ESR irradiation power = ~ 3 W, a 90 degree flip angle, ESR irradiation duration (T_ESR_)× repetition time (T_R_)× echo time (T_E_) = 0.9 × 1 × 0.04 s, number of accumulations = 1, and number of phase-encoding steps = 64. The image field of view (FOV: 32 × 32 mm) was represented by a 64 × 64 matrix. The intensity of the DNP-MR image for each sample was analyzed for a region of interest (ROI) using a software package of the Image J^50^. The ESR irradiation powers were measured using an inline peak power sensor (model MA24105A, Anritsu, Atsugi, Japan).

### Preparation of AFR samples by reaction of quinone and Asc for DNP-MR imaging

Aqueous solutions of sodium ascorbate and quinone molecules were mixed by pipetting in a 1.5-mL microtube under atmospheric conditions at room temperature. The mixture was immediately transferred to the Tefron container in the surface coil, and was set in the resonator of the DNP-MR imaging system. We finished in totally 30 s for these operations. The required time for the scan of the DNP-MR imaging to obtain one image in the above-described condition was 75 s. Thus, the scans were carried out from 30 to 105 s after the mixing of the solutions. To obtain all of the DNP-MR images in this timing, the mixture of Asc and the quinone molecule was replaced with fresh one every time after one image was obtained.

Similar operations were also performed for the ESR measurements (*e.g.*, the mixture for the measurement of the X-band ESR spectrum was transferred to the microcapillary tube and allowed to settle in the ESR resonator for 30 s). For measurements of all the ESR spectroscopies, the recorded time for the doublet AFR spectrum was not used to indicate the starting time of the measurement for whole spectrum but to indicate the displayed time for the doublet spectrum after the mixing.

### Preparation of AFR samples by reaction of Trolox radicals and Asc for DNP-MR imaging

Potassium superoxide (KO_2_) was added to ethanol solution containing 0.4 M Trolox and 0.5 M 18-Crown-6 at the weight of 0.3 mg mL^−1^ to generate Trolox radicals. After the reaction for 1 min, this Trolox radical solution was mixed with aqueous solution of 2.5 M sodium ascorbate at equal volume to produce AFR with disappearance of the Trolox radicals by pipetting. All these procedures were performed in 1.5-mL microtubes under atmospheric condition at room temperature. The radical concentrations of these Trolox and AFR radical solutions were estimated using standard curves indicating the relationships between radical concentrations and the total area in the X-band ESR spectra for methoxycarbonyl-PROXYL containing-ethanol/18-Crown-6 solutions, and mixtures of ethanol/18-Crown-6/Trolox and ^14^N-CmP aqueous solutions, respectively.

### *In vivo* DNP-MR imaging of AFR

C57BL/6N mouse was anesthetized by isoflurane, and was fixed on a holder plate. The surface coil was put on the back. After 500 μL of the aqueous mixture of Asc and CoQ_0_ was subcutaneously injected into the back, immediately, the mouse was set in resonator of the DNP-MR system, and DNP-MR imaging was performed. ICR mouse fasted for a day was anesthetized by urethane, and was fixed on a holder plate. A home-built surface coil with square-shaped single coil was put on the belly. After 500 μL of the aqueous mixture of Asc and CoQ_0_ was orally injected into the stomach with a sonde, immediately, the mouse was set in resonator of the DNP-MR system, and DNP-MR imaging was performed.

## Additional Information

**How to cite this article**: Ito, S. and Hyodo, F. Dynamic nuclear polarization-magnetic resonance imaging at low ESR irradiation frequency for ascorbyl free radicals. *Sci. Rep.* **6**, 21407; doi: 10.1038/srep21407 (2016).

## Supplementary Material

Supplementary Information

## Figures and Tables

**Table 1:** Spectroscopic DNP properties of CoQ_0_-and PQQ-mediated AFR, ^14^N-CmP, Oxo63, and FMNH.

	Radical conc. (c) (μ M)	Peak location (MHz)	Half width (MHz)	Intensity (OFF): I_0_ (a.u.)	Intensity (ON): IZ (a.u.)	Enhancement: ε	Ratio: ε/c (/μ M)
**CoQ_0_-med. AFR**	25~10 (17^A^)		198.7 (236.2**)				
Left peak		525.0	2.0 (1.8*)		844.4 (934.8**)	4.2 (4.0**)	0.2~0.4 (0.25^B^)
Right peak		520.0	1.8 (1.9*)		896.8 (911.3**)	4.5 (3.9**)	0.2~0.5 (0.27^D^)
**^14^N-CmP**	150			206.1			
Center peak		526.5	3.8		907.5	4.4	0.03
**Oxo63**	60		177.9				
Center peak		522.0	1.6		831.2	4.7	0.08
**FMNH**	120	–	–	228.1	835.7	3.7	0.03
**PQQ-med. AFR**	11~9 (10^F^)			277.5 (253.4)			
Left peak		525.0	2.0		698.2 (648.4)	2.5 (2.6)	0.2~0.3 (0.25^G^)
Right peak		520.0	2.0		642.2 (645.2)	2.3 (2.5)	0.2~0.3 (0.23^H^)

The spans of the radical concentrations and the values of ε/c for the CoQ0- and PQQ- mediated AFR were obtained with the doublet AFR spectra recorded at 30 and 105 s after the mixing (^A, F^values were obtained with the the doublet AFR spectra recorded at 60 s). ^B, D, G, H^Values are calculated with A, Fvalues, respectively. Values with no asterisk, *values, and **values of mean (n = 3) were obtained with Figs 2(b) and 1(d,f), respectively. Values for FMNH are obtained with Figure S-7d (DNP OFF and 525 MHz). Values for the PQQ-mediated AFR are from Table S-1.
