# Supplementary material for: Dynamic nuclear polarization-magnetic resonance imaging at low ESR irradiation frequency for ascorbyl free radicals
Source: Sci Rep. 2016 Feb 19;6:21407. doi: 10.1038/srep21407 (PMC4759784; doi:10.1038/srep21407)
Supplement: Supplementary Information [file srep21407-s1.pdf]

Supplementary Information

Dynamic nuclear polarization-magnetic resonance imaging at low ESR irradiation frequency for ascorbyl free radicals

Shinji Ito and Fuminori Hyodo

Innovation Center for Medical Redox Navigation, Kyushu University, Maidashi, Higashi-ku, Fukuoka 812-8582, Japan

Correspondence should be addressed to: ishinji@redoxnavi.med.kyushu-u.ac.jp

Table of Contents

Figure S-1. Production of CoQ_0-mediated AFR for DNP-MR imaging. S-2

Figure S-2. Chemical stability of CoQ_0-mediated AFR. S-3

Figure S-3. X-band, L-band, and 300-MHz ESR spectra for CoQ_0-mediated AFR. S-4

Figure S-4. DNP spectrum in the range of 471-537 MHz for ^{14}N-CmP. S-5

Figure S-5. DNP spectrum for CoQ_0-mediated AFR. S-6

Figure S-6. DNP spectra of CoQ_0-mediated AFR, ^{14}N-CmP, and Oxo63. .... S-7

Figure S-7. Production of FMNH for DNP-MR imaging. S-8

Figure S-8. Chemical stability of PQQ-mediated AFR. S-9

Figure S-9. DNP spectra for PQQ-mediated AFR, ^{14}N-CmP, and Oxo63. .... S-10

Figure S-10. Production of AFR in reaction of Asc and liposome-CoQ_{10}. S-11

Figure S-11. DNP spectra for AFR mediated by Trolox radicals. S-12

Table S-1. Spectroscoic DNP properties of the PQQ-mediated AFR. S-13

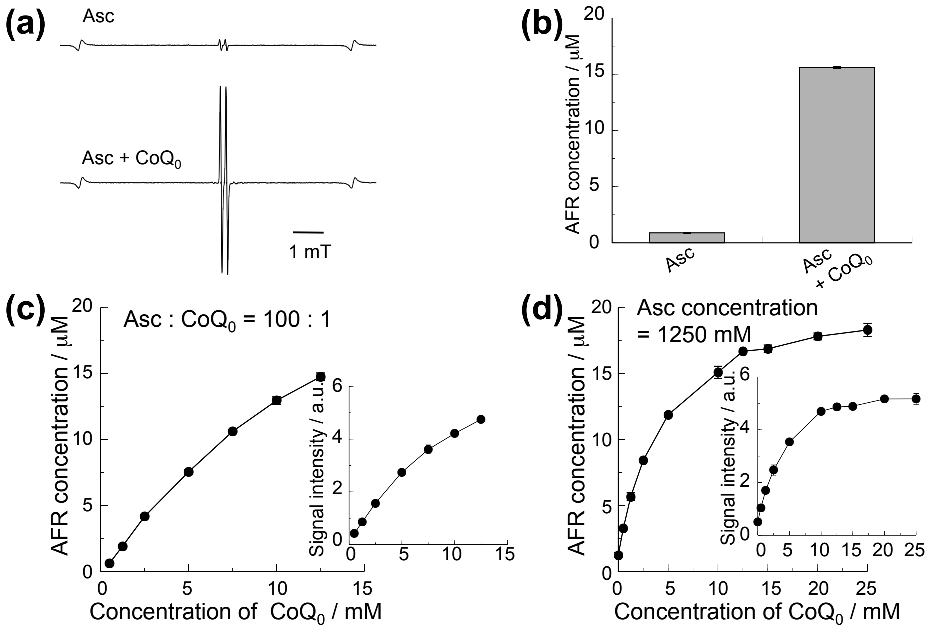

Figure S-1. Production of CoQ_0-mediated AFR for DNP-MR imaging. Typical X-band ESR spectra (a) and graphs showing the concentration of AFR produced (b) for 1250 mM Asc solution, and the mixture of 1250 mM Asc and 12.5 mM CoQ_0. In (a) and (b), equal volumes of aqueous solutions of 2500 mM sodium ascorbate and 25 mM CoQ_0 were mixed to prepare the mixture of 1250 Asc and 12.5 mM CoQ_0. (c) Relationship between concentrations of CoQ_0 and concentrations of the produced AFR when Asc and CoQ_0 solutions were mixed at a molar ratio of 100:1. (d) Relationship between the concentration of CoQ_0 and concentrations of the produced AFR when the final concentration of Asc was set to 1250 mM. In (c) and (d), horizontal axes indicate the final concentrations of CoQ_0. Insets of (c) and (d) show the variations of concentrations of CoQ_0 for the signal intensities of the doublet AFR spectra in the X-band ESR absorption spectra. The doublet spectra for the mixtures of Asc and CoQ_0 were recorded at 60 s for (a), (b) (d) and 70 s for (c) after the mixing. The data shown in (b) and (c) represent the mean values ± SD (n = 3). The data in (d) are the mean values (n = 2).

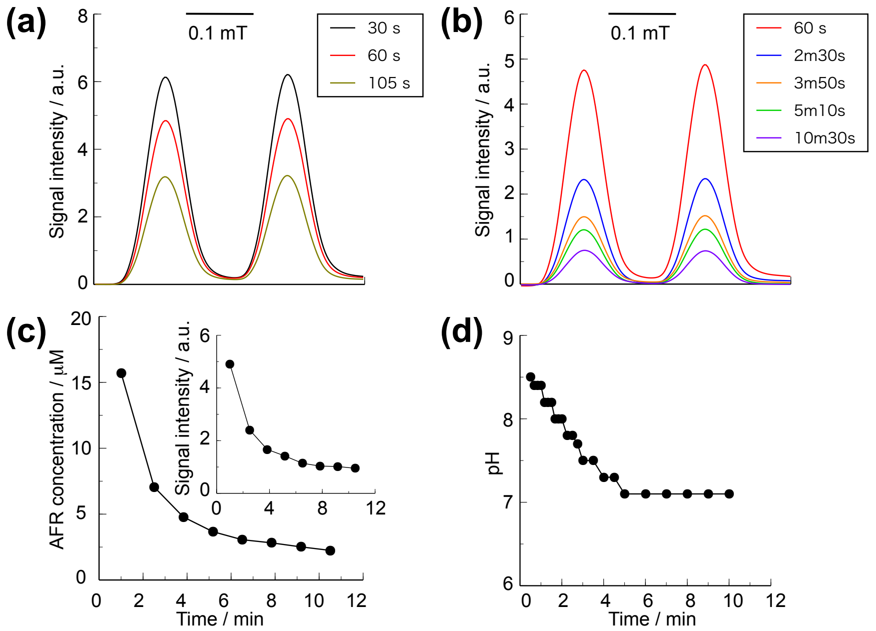

Figure S-2. Chemical stability of CoQ_0-mediated AFR. Representative X-band ESR absorption spectra obtained in various measurement times between 30 s and 105 s (a) and between 60 s and 10.5 m (b) after the mixing for the mixture of Asc and CoQ_0. Time variations in the signal intensities of the doublet spectrum (c) and the concentrations of AFR (inset), and the pH values (d) for this mixture. In (c) and (d), data were shown between 60 s and 10.5 m, and between 30 s and 10 m, respectively. In (b), (c) and (d), the variations were followed with one sample.

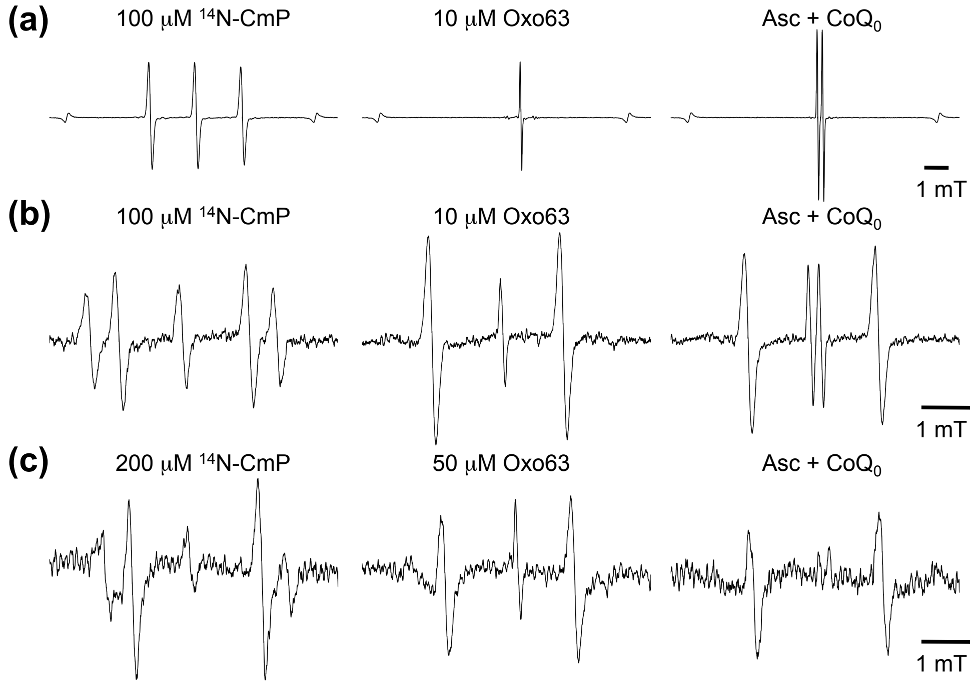

Figure S-3. X-band, L-band, and 300-MHz ESR spectra for CoQ_0-mediated AFR. Typical X-band (a), L-band (b) and 300-MHz (c) ESR spectra for the mixture of 1250 mM Asc and 12.5 mM CoQ_0, and ^{14}N-CmP and Oxo63 solutions. The concentrations of ^{14}N-CmP and Oxo63 solutions were 100 and 10 μM in (a) and (b), and 200 and 50 μM in (c), respectively. The doublet spectrum of the mixture of Asc and CoQ_0 was recorded at 60, 60, and 30 s after the mixing for the X-band, L-band, and 300-MHz ESR spectra, respectively. The ^{15}N-labeled nitroxides [^{15}N-labeled carbamoyl-PROXYL (^{15}N-CmP; purchased from Japan Redox Inc., Fukuoka, Japan) with 100 μM [in (b)] and ^{15}N-carboxy-PROXYL (^{15}N-CxP) (purchased from Japan Redox Inc.) with 400 μM [in (c)] were used as markers for the L-band and 300-MHz ESR measurements.

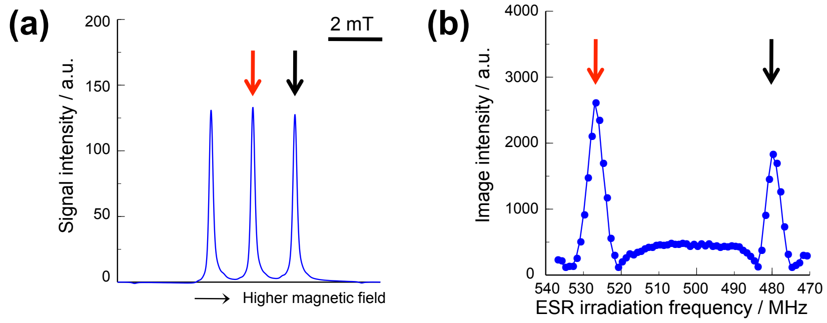

Figure S-4. DNP spectrum in the range of 471-537 MHz for ^{14}N-CmP. X-band ESR absorption (a), and DNP (b) spectra for aqueous solution of 2.5 mM ^{14}N-CmP. In (b), the DNP-MR images were obtained at every 1 MHz between 471 and 537 MHz for the 500-mL sample in a glass tube (ESR irradiation power = ~1 W; T_{ESR} = 0.5 s). The peaks at 527 and 480 MHz in the DNP spectrum correspond to the center (indicated by red arrow) and right side (indicated by black arrow) spectral peaks in the X-band absorption spectrum, respectively.

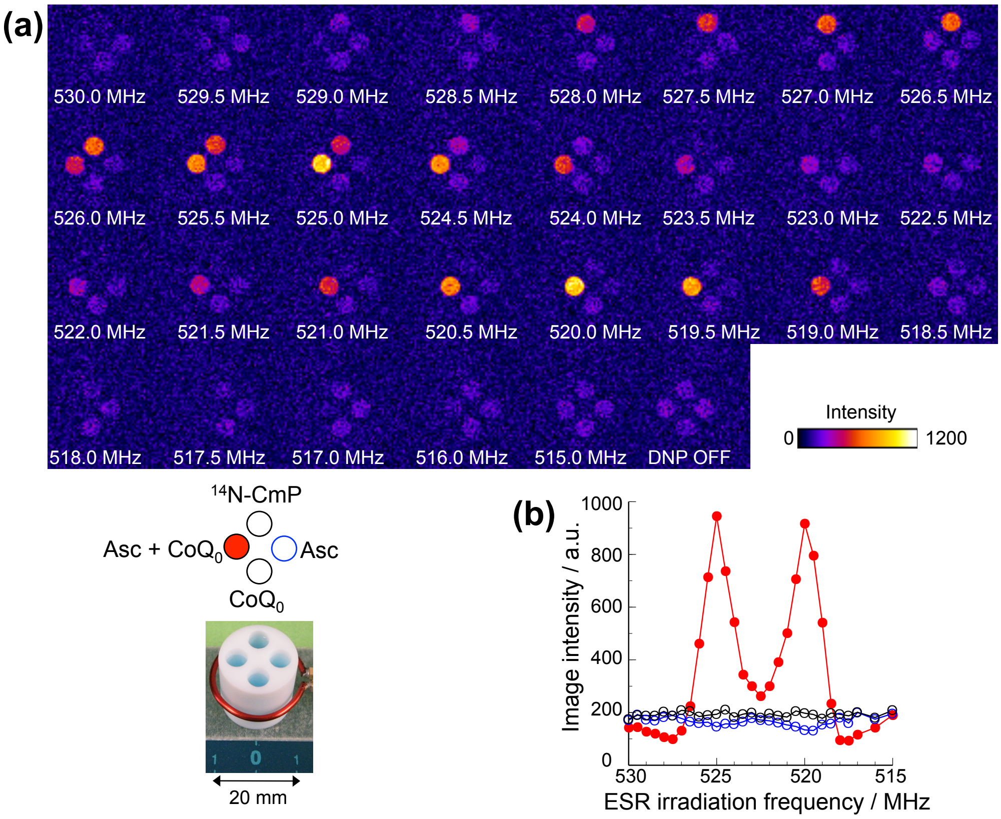

Figure S-5. DNP spectrum for CoQ_0-mediated AFR. DNP-MR images (a) and DNP spectra (b) for the mixture of 1250 mM Asc and 12.5 mM CoQ_0, and 1250 mM Asc and 12.5 mM CoQ_0 solutions. The picture in (a) shows the Teflon container. The images were obtained at every 0.5 MHz between 515 and 530 MHz.

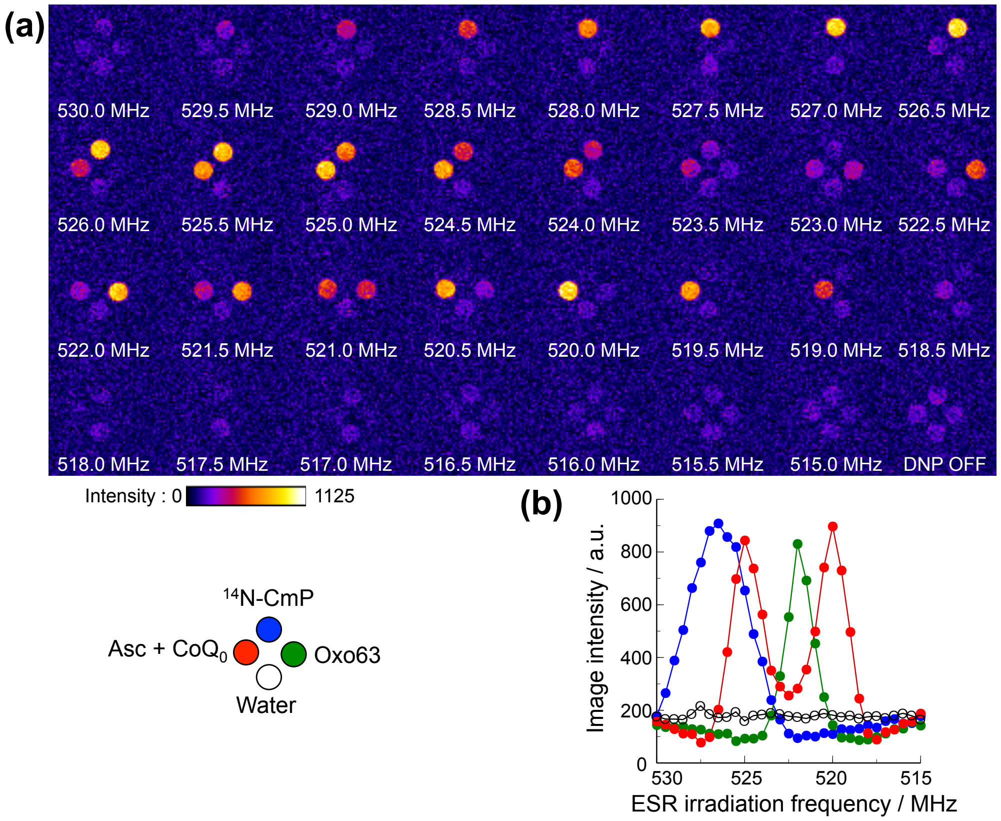

Figure S-6. DNP spectra of CoQ_0-mediated AFR, ^{14}N-CmP, and Oxo63. DNP-MR images (a) and DNP spectra (b) for the mixture of 1250 mM Asc and 12.5 mM CoQ_0, 150 μM ^{14}N-CmP and 60 μM Oxo63 solutions, and ultrapure water. The images were obtained at every 0.5 MHz between 515 and 530 MHz.

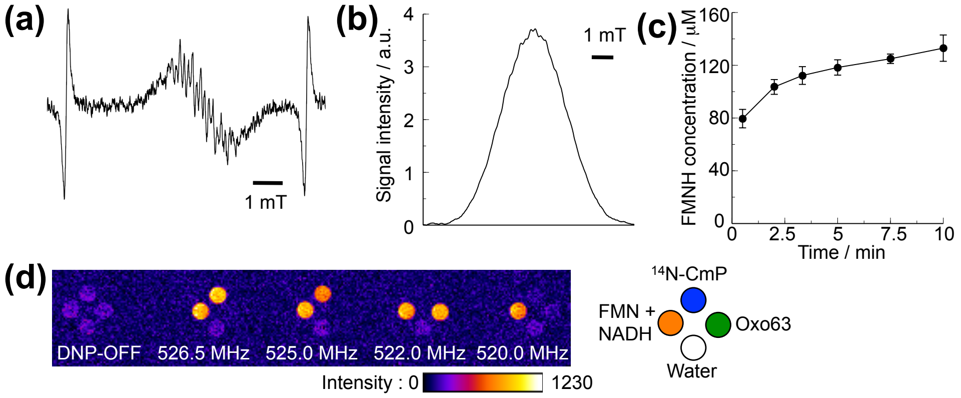

Figure S-7. Production of FMNH for DNP-MR imaging. Typical X-band ESR (a) and the absorption (b) spectrum for the mixture of 9 mM FMN and 9 mM NADH. Aqueous solutions of 18 mM FNN and 18 mM NADH were mixed at the equal volume at room temperature under atmospheric conditions. X-band ESR spectrum and its absorption spectrum for this mixture indicated the production of FMNH (c) Time variation in the FMNH concentration after the mixing. (d) DNP-MR images for the mixture of FMN and NADH, 150 μM ^{14}N-CmP and 60 μM Oxo63 solutions, and ultrapure water. In (a) and (d), the mixture was used 7.5 m after the mixing. In (b), the absorption spectrum is obtained by integration of the spectrum shown in (a). The data shown in (c) represent the mean values ± SD (n = 5).

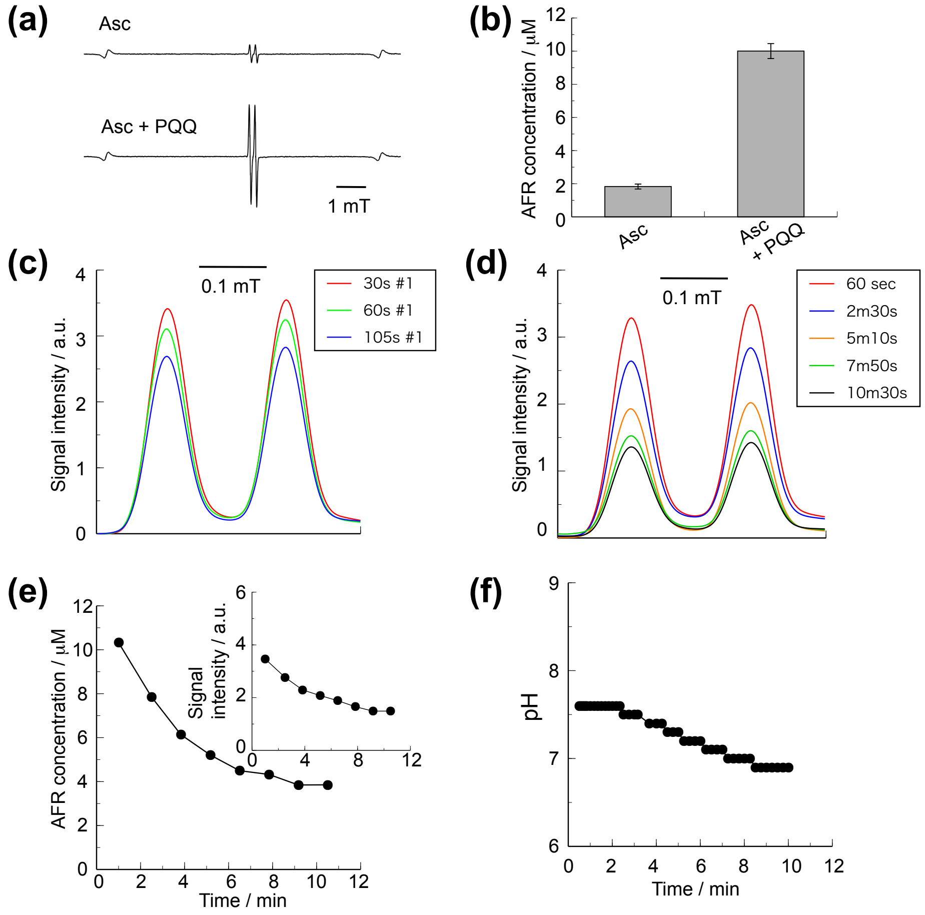

Figure S-8. Chemical stability of PQQ-mediated AFR. Typical X-band ESR spectra (a) and graphs of the concentration of AFR (b) for the 2125 mM Asc solution, and the mixture of 2125 mM Asc and 4.5 mM PQQ. Representative time variations in the doublet AFR spectrum (c) and the signal intensities (d) in the measurements of the X-band ESR absorption spectra for the mixture of Asc and PQQ between 60 s and 10.5 m after the mixing. Aqueous solutions of 2500 mM sodium ascorbate and 30 mM PQQ were mixed at volume ratio of 17:3 at room temperature under atmospheric conditions to prepare the mixture of 2125 mM Asc and 4.5 mM PQQ. Time variations in the concentrations of AFR (e) and the pH values (f) for this mixture after in this time range. The spectra shown in (c) are obtained by integration of the spectrum in (a) and its time variation. The variations were followed with one sample. The data in (b) represent the mean values ± SD (n = 3).

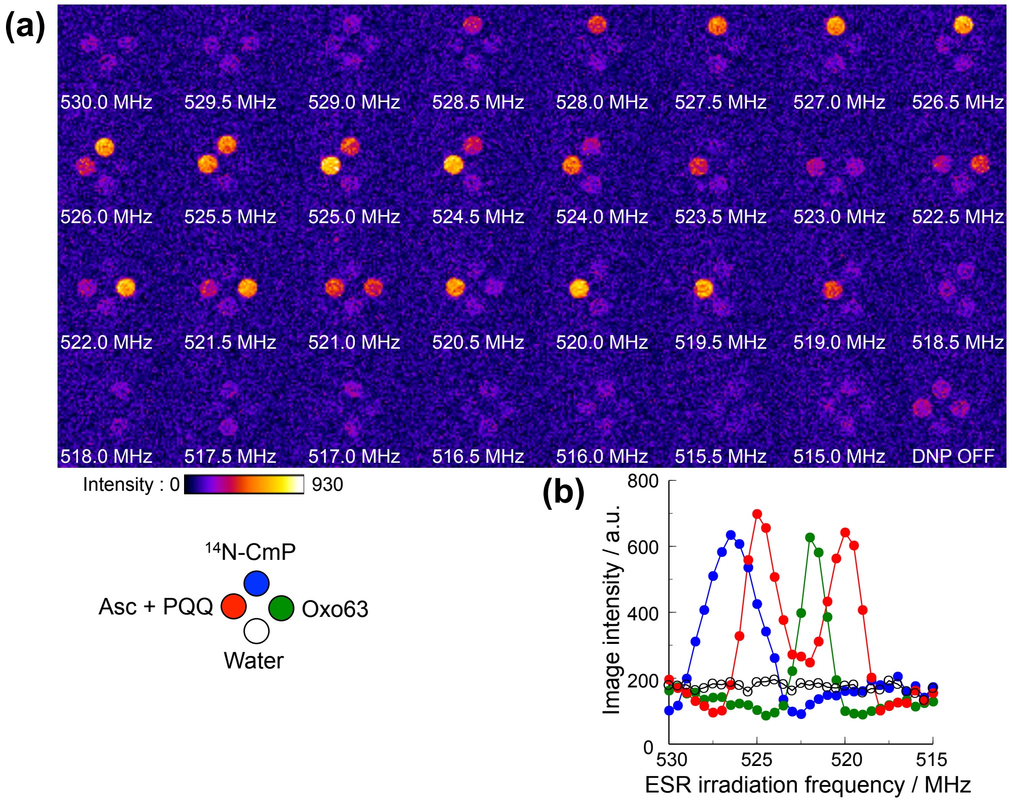

Figure S-9. DNP spectra for PQQ-mediated AFR, ^{14}N-CmP, and Oxo63. DNP-MR images (a) and DNP spectra (b) for the mixture of 2125 mM Asc and 4.5 mM PQQ, 90 μM ^{14}N-CmP, 50 μM Oxo63 solutions, and ultrapure water. The images were obtained at every 0.5 MHz between 515 and 530 MHz.

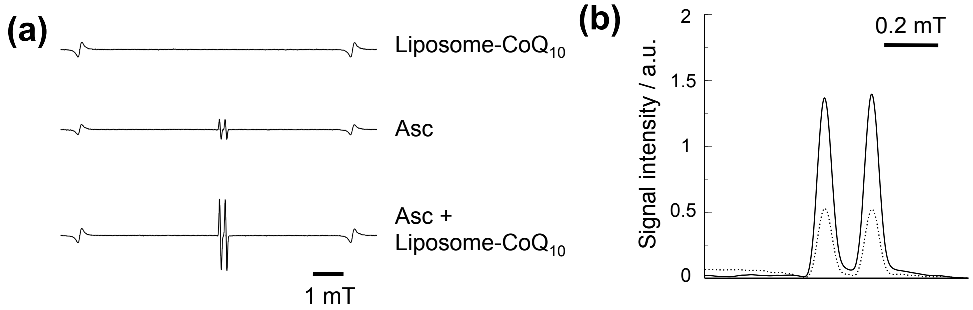

Figure S-10. Production of AFR in reaction of Asc and liposome-CoQ$_{10}$. Typical X-band ESR spectra for a CoQ$_{10}$-containing liposome suspension, 625 mM Asc solution, and the mixture of 625 mM Asc solution and CoQ$_{10}$-containing liposome suspension. Sodium ascorbate solution (2500 mM) and a CoQ$_{10}$-containing liposome aqueous suspension [0.1 g of CoQ$_{10}$-containing liposome powder (Presome Q$_{10}$; gifted from NOF Corporation, Tokyo, Japan) was suspended in 500 μL of 50 mM Tris-HCl buffer (pH 8.8)] were mixed at the ratio of 1:3 under atmospheric conditions at room temperature (the final concentration of Asc was 625 mM). The doublet spectrum for the mixture was recorded at 70 s after the mixing.

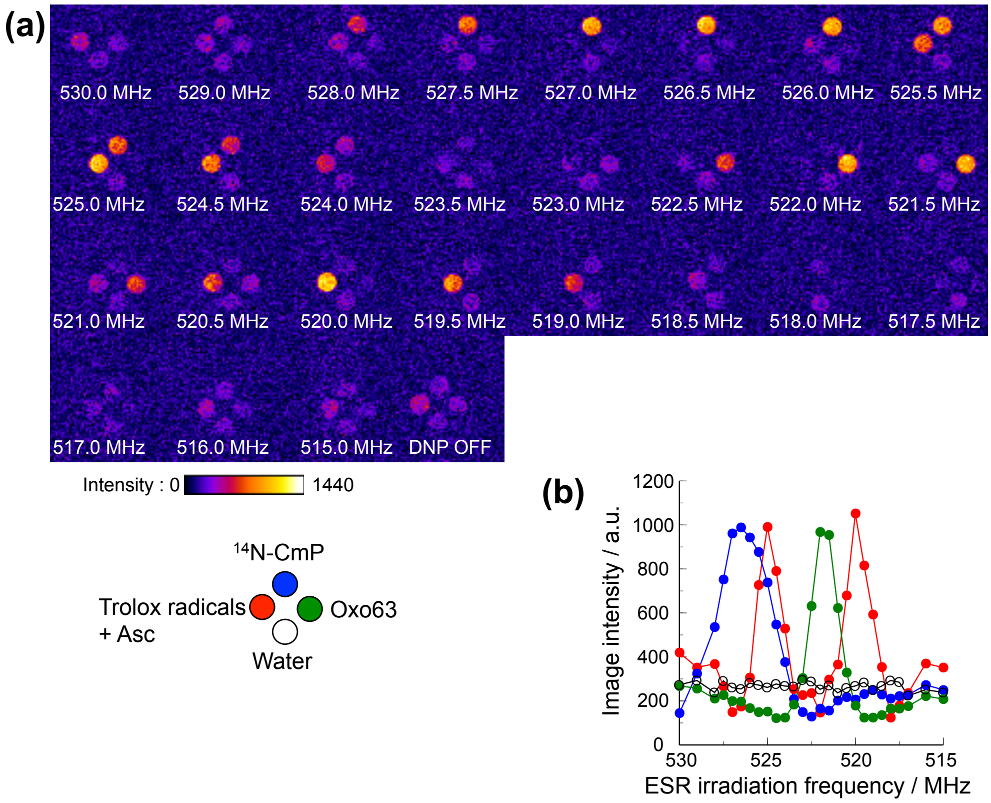

Figure S-11. DNP spectra for AFR mediated by Trolox radicals, ^{14}N-CmP, and Oxo63. DNP-MR images (a) and DNP spectra (b) for the mixture of Asc and Trolox radicals, 100 μM ^{14}N-CmP, 50 μM Oxo63 solutions, and ultrapure water. The images were obtained at every 0.5 MHz between 515 and 530 MHz.

Table S-1. Spectroscopic DNP properties of the PQQ-mediated AFR.

	Radical conc.(c) (μM)	Peak location (MHz)	Half width (MHz)	Image Intensity I_0 (a.u.)	I_Z (a.u.)	Enhancement ε	ε/c (/μM)
PQQ-med. AFR	11 ~ 9 (10[F])			277.5 (253.4*)			
Left peak		525.0	2.0		698.2 (648.4*)	2.5 (2.6*)	0.2 ~ 0.3 (0.25[G])
Right peak		520.0	2.0		642.2 (645.2*)	2.3 (2.5*)	0.2 ~ 0.3 (0.23[H])
^{14}N-CmP	90			233.2			
Center peak		526.5	3.2		634.0	2.7	0.03
Oxo63	50			225.6			
Center peak		522.0	1.5		627.0	2.8	0.06

The spans of the radical concentrations and the values of ε/c for the PQQ-mediated AFR were obtained with the doublet AFR spectra recorded at 30 and 105 s after the mixing ([F] value was obtained with the the doublet AFR spectrum recorded at 60 s). [G, H] Values are calculated with [F] value. Values with no asterisk and * values of mean (n = 3) were obtained with Figures 4d (Figure S-9b) and 4f, respectively.
